# Emergence of Distinct *Salmonella enterica* Serovar Enteritidis Lineage since 2020, South Korea

**DOI:** 10.3201/eid3107.250043

**Published:** 2025-07

**Authors:** Eunkyung Shin, Tae-Min La, Jaeil Yoo, Junyoung Kim, Ji-Yeon Hyeon

**Affiliations:** Korea Centers for Diseases Control and Prevention Agency, Cheongju, South Korea (E. Shin, J. Yoo, J. Kim); Konkuk University College of Veterinary Medicine, Seoul, South Korea (T.-M. La, J.-Y. Hyeon)

**Keywords:** *Salmonella enterica* serovar Enteritidis, *Salmonella* Enteritidis, salmonellosis, outbreak, whole-genome sequencing, whole-genome single-nucleotide polymorphism, SNP, phylogeographic analysis, antimicrobial resistance, bacteria, enteric infections, South Korea

## Abstract

We analyzed whole-genome sequences of *Salmonella enterica* serovar Enteritidis isolates in South Korea that had the SEGX01.049 pulsed-field gel electrophoresis pattern. That lineage has emerged and circulated in South Korea since 2020, leading to 2 fatal infection cases. We investigated the genomic characteristics and identified potential sources of that lineage. Isolates from outbreaks during 2020–2023 clustered in the Global IIa clade, along with other *Salmonella* Enteritidis strains from chicken farms in South Korea and human isolates from the United Kingdom. Bayesian molecular clock analysis estimated the time to the most recent common ancestor of our isolates in the Global IIa clade was 2017.57. Moreover, phylogeographic analysis supported substantial statistical evidence (Bayes factor 111.415; posterior probability 0.97) for the introduction of this lineage into South Korea from the United Kingdom. Continued genomic surveillance will be needed to monitor the spread of foodborne pathogens such as *Salmonella* Enteritidis and improve prevention strategies.

*Salmonella enterica* serovar Enteritidis is a major pathogen responsible for human salmonellosis worldwide; substantial public health implications exist because of its rapid transmission and frequent association with foodborne outbreaks (*1*). In South Korea, *Salmonella* Enteritidis has emerged as a leading cause of foodborne illness and one of the most prevalent bacterial pathogens (*2*,*3*). Poultry products, especially eggs, have been consistently identified as primary sources of transmission; recent severe outbreaks have led to fatal salmonellosis cases (*4*). That trend highlights the critical need for comprehensive surveillance systems and effective outbreak response strategies to control *Salmonella* Enteritidis infections.

In South Korea, surveillance of *Salmonella* Enteritidis has substantially transitioned within the PulseNet Korea network, part of a global network designed to detect and investigate foodborne infections. PulseNet Korea has evolved from reliance on pulsed-field gel electrophoresis (PFGE) to the use of whole-genome sequencing (WGS) to track genomic patterns and trace infection sources. Since the nationwide implementation of WGS in 2020, more detailed analyses of genetic diversity and phylogenetic relationships among strains exhibiting identical PFGE patterns has become possible. High-resolution typing methods, such as whole-genome single-nucleotide polymorphism (SNP) analysis and core-genome multilocus sequence typing (cgMLST), have become standard for pathogen surveillance, including for *S*. *enterica* (*5*–*7*). Furthermore, cgMLST data are commonly analyzed by using the HierCC hierarchical clustering package, a threshold-based method grouping isolates by allelic differences, implemented in the EnteroBase web-based platform (*7*,*8*). Although previous studies in South Korea have applied WGS to *Salmonella* Enteritidis strains isolated from poultry and food sources (*9*,*10*), research focusing on human *Salmonella* Enteritidis isolates remains limited.

We analyzed whole-genome sequences of *Salmonella* Enteritidis isolates with the SEGX01.049 PFGE pattern submitted to the Korea Disease Control and Prevention Agency (KDCA); isolates with that pattern have emerged and circulated in South Korea since 2020 and have caused 2 fatal salmonellosis cases. Two foodborne outbreaks associated with that PFGE pattern were reported in Gyeongnam Province, South Korea, in 2021 (*11*). Despite repeated detection in outbreak investigations, limited genomic data have been available to contextualize that *Salmonella* subtype within a global framework. Therefore, we investigated the genomic characteristics and phylogenetic relationships of those isolates and compared them with *Salmonella* Enteritidis strains from other global regions to identify potential food sources and trace the origins of this subtype in South Korea. No ethics approvals were required for this study.

## Materials and Methods

### Bacterial Isolates

Member laboratories of national gastrointestinal infection disease surveillance networks (EnterNet and PulseNet Korea) collected and anonymously submitted *Salmonella* spp. isolates to KDCA. We identified the bacteria by using a VITEK-II automated system (bioMérieux, https://www.biomerieux.com). We performed *Salmonella* serotyping according to the Kauffmann-White scheme using specific antiserum (BD Biosciences, https://www.bdbiosciences.com). We analyzed isolates by using PFGE after DNA restriction enzyme digestion with *Xba*I in accordance with the PulseNet International protocol (https://www.pulsenetinternational.org). We determined genetic similarities between PFGE patterns by using BioNumerics version 7.5 (bioMérieux). For WGS, we selected 38 SEGX01.049 *Salmonella* Enteritidis isolates collected from patients, workers, the environment, and food associated with 3 sporadic infection cases in 2013 and 2014 and 8 outbreaks during 2020–2023 (Appendix 1 Table 1).

### Whole-Genome Sequence Analysis

We isolated genomic DNA by using a DNA Blood and Tissue Kit (QIAGEN, https://www.qiagen.com). We performed WGS on the MiSeq platform by using the v2 kit (500 cycles, 2 × 250-nt reads) (Illumina Inc., https://www.illumina.com). We adaptor-trimmed raw reads by using Bbduk (https://sourceforge.net/projects/bbmap) and used a quality threshold of Q>20 and a minimum length of 50 bp. We de novo assembled trimmed reads by using SPAdes 3.15.5 (*12*) with default settings in Geneious Prime 10 (https://www.geneious.com). We determined the presence of acquired antimicrobial resistance genes by using ABRicate version 1.0.1 (https://github.com/tseemann/abricate) and the ResFinder database (*13*) and determined virulence genes by using default settings in the virulence factor database (*14*). We identified prophages by using PHASTEST (*15*). We deposited paired-end reads of *Salmonella* Enteritidis isolates from this study into the National Center for Biotechnology Information (NCBI; https://www.ncbi.nlm.nih.gov) Bioproject database (accession no. PRJNA1150652).

### Phylogenetic Analysis

We constructed 2 whole-genome SNP (wgSNP) phylogenetic trees: 1 tree to compare the 38 isolates from this study with 223 *Salmonella* Enteritidis isolates from South Korea and 1 tree to compare the 38 isolates with 1,230 *Salmonella* Enteritidis isolates, which included the 223 genomic sequences from South Korea, 93 selected sequences from the HC5_2301 cluster (*7*), and 914 sequences reported previously (*16*). We compiled genomic sequences of *Salmonella* Enteritidis isolates from South Korea (n = 223) from multiple sources (Appendix 1 Table 2). We retrieved 118 genome sequences from GenBank and EnteroBase (https://enterobase.warwick.ac.uk) public databases by using the search terms “*Salmonella* Enteritidis” and “South Korea” for GenBank and “Enteritidis” (serovar) and “South Korea” (country) for Enterobase (both databases accessed January 2024). We identified and excluded duplicate or redundant entries across the 2 databases. We obtained an additional 24 genomes from our previous study (*9*); those were not indexed under the “*Salmonella* Enteritidis” in GenBank and, thus, could not be retrieved by using standard search terms. Furthermore, 81 genomes were provided by KDCA and Konkuk University, Seoul, South Korea. We identified wgSNPs for the downloaded sequences and 38 *Salmonella* Enteritidis isolates from this study by using Snippy version 4.6.0 (https://github.com/tseemann/snippy); we used *Salmonella* Enteritidis strain P125109 (GenBank accession no. NC011294) as the reference genome. We identified prophages in the reference genome by using PHASTEST (*15*). We identified repetitive regions by aligning the reference genome to itself by using the NUCmer commands in the MUMmer package version 3.23 (*17*) along with the --maxmatch and --nosimplify options. We predicted recombination regions by using Gubbins version 3.3.1 (*18*) and defined repetitive regions by using the default threshold of >20 bp; the shortest masked region in this study was 68 bp. We excluded prophage, repetitive, and recombinant regions, and the final alignment had 4,326 SNP sites for the dataset from Korea and 4,591 SNP sites for the global dataset. We constructed a maximum-likelihood phylogenetic tree from the clean alignment by using a transversion model plus empirical base frequencies, selected by ModelFinder in IQ-TREE (*19*), with 1,000 bootstrap replicates. We analyzed the population structure of *Salmonella* Enteritidis isolates by using default parameters in FastBAPS (*20*).

For global phylogenetic analysis, we retrieved 914 whole-genome sequences of *Salmonella* Enteritidis used in a previous study (*16*) from the NCBI SRA database (https://www.ncbi.nlm.nih.gov/sra) according to their accession numbers by using fasterq-dump from the SRA Toolkit (https://github.com/ncbi/sra-tools); those sequences represented epidemiologic, geographic, and phylogenetic diversity. In addition, we downloaded 3,609 genomic sequences in the cgMLST HC5_2301 cluster from EnteroBase (*8*), because the isolates from this study clustered with *Salmonella* Enteritidis strain MFDS1018147 (GenBank accession no. CP110220.1) within that cluster. We constructed a neighbor-joining tree by using Mashtree version 1.4.6 (*21*) according to Mash distance; the tree included the 3,609 cgMLST HC5_2301 genomes and 261 isolates from South Korea (including the 38 isolates from this study). From this tree, we selected 93 of the 3,609 sequences showing close Mash distance proximity to the isolates in this study for wgSNP analysis. (Appendix 1 Table 2, Figure). For wgSNP analysis, we used a total of 1,268 sequences, comprising the isolates from this study (n = 38), those from the cgMLST HC5_2301 cluster (n = 93), sequences used in a previous study (n = 914) (*16*), and other *Salmonella* Enteritidis isolates from South Korea (n = 223), and we determined the population structure by using FastBAPS (*20*).

### Bayesian Phylogenetic Analysis

**Figure 1:**
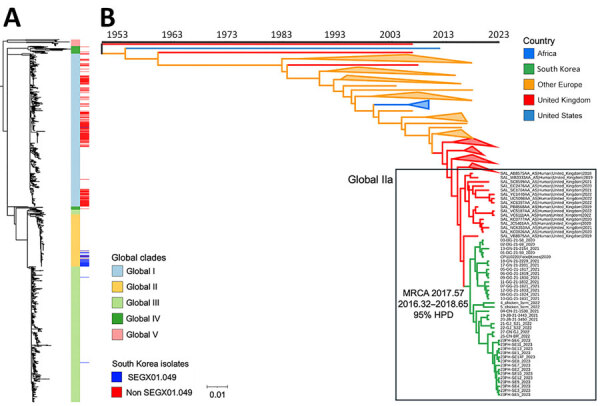
Phylogenetic analysis of *Salmonella enterica* serovar Enteritidis global lineages and those from South Korea. A) Maximum-likelihood phylogenetic tree of whole-genome single-nucleotide polymorphisms of 1,268 global *Salmonella* Enteritidis isolates. Global clades separated by using FastBAPS (*20*) and isolates from South Korea are indicated. Scale bar indicates number of single-nucleotide polymorphisms per site. B) Time-scaled maximum clade credibility tree of the Global II clade of *Salmonella* Enteritidis. Box shows Global IIa clade sequences from South Korea and the United Kingdom; inferred median MRCA and 95% HPD interval are indicated for isolates from South Korea. HPD, highest posterior density; MRCA, most recent common ancestor.

To reconstruct the evolutionary history, we aligned wgSNPs sequences (n = 180) within the FastBAPS Global II clade from the phylogenetic tree of 1,268 sequences (Figure 1, panel A). Among 185 sequences in the FastBAPS Global II clade, we excluded 1 wild animal sample from Germany, 1 from the United Kingdom, and 1 isolate labeled as ND from the United Kingdom within the cgMLST HC5_2301 cluster because of ambiguous host or source metadata, including undefined host or sampling origin. In addition, we excluded sequences from Vietnam and Australia (n = 1 each) reported in the previous study (*16*) because of their limited representation.

We explored the temporal signal (*R*^2^>0.686) for molecular clock analysis of the phylogenetic tree by using TempEst version 1.5.3 (*22*). We performed Bayesian phylogenetic analysis by using BEAST version 1.10.4 (*23*). We selected the transversion nucleotide substitution model with empirical base frequencies by using ModelTest-NG (*24*). To identify the best-fit model for the data, we compared the marginal likelihood of 2 clock models (strict and lognormal relaxed) in combination with 3 tree priors (constant population size, exponential growth, and Bayesian skyline) by using path sampling and stepping-stone sampling (*25*). The lognormal relaxed clock model with a constant population size prior consistently yielded the highest Bayes factor, indicating the best fit for our dataset.

For Bayesian discrete trait phylogeographic analysis, we reconstructed ancestral locations and estimated asymmetric exchanges between regions by using a nonreversible, continuous-time Markov chain model. To minimize potential sample size bias, we grouped sequences from regions with low representation into broader categories according to geographic relevance. The Africa group had 11 sequences from Mauritius (n = 3), Côte d’Ivoire (n = 1), Uganda (n = 2), and South Africa (n = 5). The Other Europe group comprised 25 sequences from Austria (n = 3), Belgium (n = 2), Denmark (n = 3), Finland (n = 1), Germany (n = 3), Italy (n = 5), Poland (n = 7), and Sweden (n = 1). The United Kingdom group was the largest group with 97 sequences, followed by South Korea with 38 sequences and the United States with 10 sequences.

We applied a Bayesian stochastic search variable selection procedure to identify well-supported transitions between discrete states by using the Bayes factors test in SPREAD3 software version 0.9.6 (*26*). We considered transitions to be significant when the posterior probability was >0.5 and the Bayes factor was >3 (*26*). We ran 3 independent chains of 100 million generations in parallel, sampling every 10,000 generations. We evaluated the results in TRACER version 1.7.1 (http://beast.community/tracer) to ensure that the effective sample sizes were >200 and to ensure convergence between all 3 runs. We combined the log and tree files of 3 independent runs by using LogCombiner version 1.8.3 (https://beast.community/logcombiner) and 10% burn-in. We generated a maximum clade credibility tree by using TreeAnnotator version 1.8.2 (https://beast.community/treeannotator) and 10% burn-in and common ancestor node heights. We used Interactive Tree of Life version 6 (https://itol.embl.de) to annotate the tree.

To evaluate the potential effect of sampling bias, we constructed a generalized linear model as an extension of phylogeographic inference by using BEAST. That analysis assessed the influence of source and sink sample sizes as predictors on migration rates within the Bayesian framework. Coefficients quantified the effect size, whereas indicators determined predictor inclusion, which we analyzed by using TRACER. We used sample sizes for each discrete state as predictors to inform transition rate estimates.

## Results

### Prevalence of SEGX01.049 PFGE Patterns

We determined the 10 most prevalent PFGE patterns of *Salmonella* Enteritidis isolates from sporadic cases and outbreaks submitted to the KDCA during 2013–2024 (Appendix 1 Table 3). The frequency of SEGX01.049 isolates markedly increased from 3.2% in 2018 to 96.5% by August 2024; in addition, the percentage of SEGX01.049-related outbreaks rose from 33.3% in 2020 to 91.7% in 2024 (Appendix 1 Table 3). Among the 23 reported SEGX01.049 outbreaks during 2020–2024, 13 (56%) outbreaks were caused by egg-associated foods, 9 from an unknown source, and 1 from another food type (Appendix 1 Table 4).

### Phylogenetic Analysis of *Salmonella* Enteritidis Isolates

**Figure 2:**
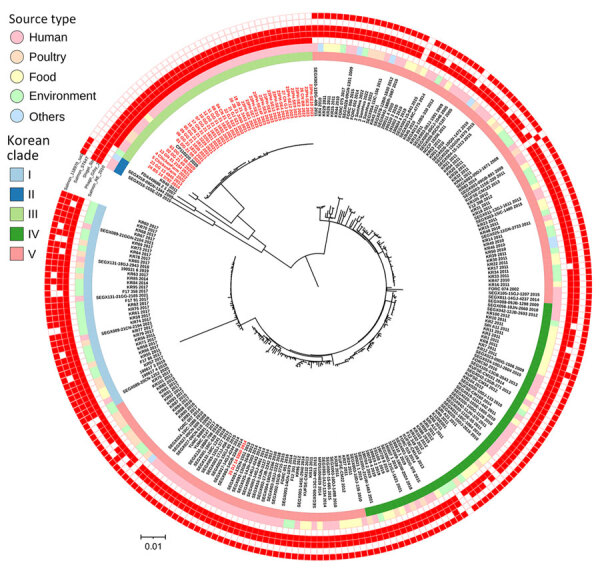
Phylogenetic analysis of distinct *Salmonella* *enterica* serovar Enteritidis lineage since 2020, South Korea. Maximum-likelihood phylogenetic tree was constructed for whole-genome single-nucleotide polymorphisms of *Salmonella* Enteritidis from South Korea (n = 223) and isolates sequenced in this study (n = 38). Red text indicates isolates from this study. Innermost ring indicates clades separated by using FastBAPS (*20*), followed by sources of isolates. Five outer rings indicate the presence of different prophages; red boxes indicate the presence of prophages with a total count of >30. Isolation year is indicated at the end of each isolate number. Scale bar indicates number of single-nucleotide polymorphisms per site.

We constructed 2 wgSNP phylogenetic trees to compare the isolates from this study with 223 other *Salmonella* Enteritidis genome sequences from South Korea or 1,230 global *Salmonella* Enteritidis genome sequences. The genomes from South Korea grouped into 5 clades (Korea I–V) (Figure 2). All 38 isolates in this study grouped within the distinct Korea III clade, except for isolate 25-DJ-24–5950, which was obtained from a sporadic case in 2014 and grouped within the Korea V clade (Figure 2; Appendix 1 Table 1). Among the 223 *Salmonella* Enteritidis genome sequences from South Korea, 3 sequences from poultry sources (4_chicken farm, 5_chicken farm, and KR40) and 1 sequence (CP11020.1) from food associated with an outbreak (braised burdock) grouped within the Korea III clade (Figure 2). Other genome sequences from poultry sources clustered in Korea I, II, IV, and V clades, indicating a divergence from the isolates in the Korea III clade.

In the phylogenetic tree comprising 1,268 global *Salmonella* Enteritidis sequences (Figure 1, panel A), the sequences grouped into 5 clades (Global I–V); isolates from South Korea grouped into Global I–IV clades. All outbreak-associated isolates collected during 2020–2023 in this study (n = 35) grouped in the Global II clade, along with 3 *Salmonella* Enteritidis genome sequences (CP11020.1, 4_chicken farm, and 5_chicken farm) from South Korea, all isolates selected from the HC5_2301 cluster, and isolates from the Atlantic lineages described previously (*16*) (Appendix 1 Table 1). Three isolates from sporadic cases in 2013 and 2014 in this study belonged to Global I and Global III clades (Appendix 1 Table 1).

### Phylogeographic Analysis of Isolates from Other Countries

We performed Bayesian phylogenetic analysis to reconstruct the temporal history of the Global II clade (Figure 1, panel A). The time to most recent common ancestor of isolates from South Korea, including the isolates from outbreaks during 2020–2023 (4_chicken farm, 5_chicken farm, and CP110220.1), was estimated as 2017.57 (95% credible interval 2016.32–2018.65) (Figure 1, panel B). The isolates from South Korea within the Global II clade shared recent common ancestors with human isolates (n = 16; Global IIa clade) from the United Kingdom (Figure 1, panel B).

Bayesian phylogeographic analysis of the Global II clade strongly supported the most likely introduction of *Salmonella* Enteritidis into South Korea from the United Kingdom (Bayes factor 111.414; posterior probability 0.97), suggesting the most probable source of this distinct lineage (Appendix 1 Table 5). Other transmission routes were also possible (Appendix 1 Table 5). The generalized linear model analysis indicated sample size had minimal influence on introducing potential bias toward the origin or destination state in the analysis (source indicator, 1.555 × 10^–3^; source coefficient, 4.346 × 10^–3^; sink indicator, 8.88 × 10^–4^; and sink coefficient, 2.48 × 10^–2^).

### Prophage Patterns, Antimicrobial Drug Resistance, and Virulence Gene Profiles

We summarized the prophage pattern, antimicrobial drug resistance, and virulence gene profiles of the isolates in this study, along with the isolates from the United Kingdom that shared recent common ancestors with our isolates in the Global IIa clade (Appendix 1 Table 6; Appendix 2 Tables 1, 2). We identified 21 prophages in the isolates from South Korea and those in the Global IIa clade (Figure 2; Appendix 1 Table 6). All isolates within the Global IIa clade exhibited a distinct prophage pattern, harboring Shigel Stx, Phage_Gifsy_2, and Salmon_RE_2010; the Salmon_RE_2010 prophage pattern was absent in other isolates from Korea (Figure 2; Appendix 1 Table 6). All isolates from South Korea carried the *aac(6′)-Iaa* aminoglycoside resistance gene. All isolates in the Global II clade, except for 1 isolate (4_chicken farm), exhibited an identical antimicrobial drug resistance profile; the *aac(6′)-Iaa* gene was their sole antimicrobial drug resistance determinant (Appendix 2 Table 1). We identified 115 virulence genes in the isolates (Appendix 2 Table 2). The isolates from this study, along with those from the United Kingdom within the Global IIa clade, exhibited similar virulence gene profiles. All isolates carried the complete set of 115 virulence genes, except for *gogB* and *ssek2*, and were divided into 2 distinct profiles according to the presence or absence of the *shdA* gene.

## Discussion

Nontyphoidal *Salmonella* serovars generally cause self-limiting gastroenteritis in humans. However, in industrialized countries, <5% of cases can progress to invasive extraintestinal disease, leading to bacteremia and focal systemic infections (*27*). Despite the 2 fatal cases associated with the SEGX01.049 type *Salmonella* Enteritidis strains in the Korea III clade, the antimicrobial drug resistance and virulence gene profiles of the fatality-linked isolates from this study were not substantially different from those of other *Salmonella* Enteritidis isolates in South Korea. According to clinical data submitted to KDCA, both fatal cases occurred in patients without documented underlying health conditions. Although host-related factors are known to considerably affect disease outcomes, those clinical findings raise the possibility that pathogen-specific factors, potentially located in noncoding or regulatory regions of the bacterial genome, might also contribute to clinical outcomes. Furthermore, the HC5_2301 cluster has been associated with fatal cases and elevated hospitalization rates in a multicountry outbreak in Europe (*5*).

Phylogenetic analyses suggested that SEGX01.049 *Salmonella* Enteritidis isolates from outbreaks during 2020–2024 formed a distinct group, separate from other isolates from South Korea, and shared a recent common ancestor with human Global IIa clade isolates from the United Kingdom. Moreover, the human isolates within the Global IIa clade exhibited similar prophage patterns, antimicrobial drug resistance profiles, and virulence gene profiles, further supporting the hypothesis of potential introduction of those strains into South Korea. The estimated time to most recent common ancestor of isolates from South Korea within the Global IIa clade was 2017.57, suggesting that introduction into South Korea occurred ≈5 months before the first detection of the SEGX01.049 PFGE pattern in October 2018.

Global dissemination of *Salmonella* Enteritidis has been driven by centralized poultry breeding and international trade (*16*). On the basis of genome sequences and their metadata used in our analysis, it was not possible to determine whether the SEGX01.049 strain was introduced into South Korea via poultry products, trade, or infected travelers. Data from the Food and Agriculture Organization of the United Nations indicated that an average of ≈177,000 live chickens per year were imported into South Korea from the United Kingdom during 2015–2020; the average annual declared value was ≈$3.26 million US (*28*). Although the specific breed and production purpose of live chickens imported from the United Kingdom are not disclosed in those data, such shipments typically involve breeding stock. Given the central role of imported breeding stock in South Korea’s poultry industry, this trade route represents a plausible pathway for the introduction of the SEGX01.049 type *Salmonella* Enteritidis lineage. After the introduction, poultry products played a substantial role in dissemination of that strain, leading to foodborne outbreaks. We showed that 12 of 23 SEGX01.049 outbreaks during 2020–2024 were caused by contaminated eggs (Appendix 1 Table 4), and outbreak-related isolates sequenced in this study, which included samples from human, food, and environmental sources, phylogenetically clustered with *Salmonella* Enteritidis strains from chicken farms in South Korea (Figure 1); those data support the association between SEGX01.049 outbreaks in Korea and domestically produced poultry products. Consistent with that finding, *Salmonella* Enteritidis isolates within the HC5_2301 cluster have been linked to multiple foodborne illness outbreaks associated with the consumption of chicken meat or eggs across Europe since 2014 (*5*,*6*,*29*).

The first limitation of our study is that the analyses relied on retrospective data, which might not capture all instances of SEGX01.049 strains. Second, the genomic analysis was constrained by the availability of reference genomes. Future research should focus on expanding genomic studies to include more diverse isolates and regions. Investigating the role of specific genetic elements in pathogenicity and exploring environmental factors influencing the prevalence of SEGX01.049 would provide valuable insights. Moreover, studies on the potential effects of preventive measures and interventions in controlling outbreaks would be beneficial.

In conclusion, we provide insights into the phylogenetic relationships and potential introduction routes of *Salmonella* Enteritidis into South Korea. Enhanced biosecurity is required to prevent the introduction and dissemination of *Salmonella* Enteritidis across the poultry industry; the role that human activity can play in spread should not be underestimated. Continued genomic surveillance remains invaluable to monitor the spread of foodborne pathogens; such efforts could further the design of improved prevention strategies.

Appendix 1 Additional information for emergence of distinct *Salmonella enterica* serovar Enteritidis lineage since 2020, South Korea.

Appendix 2 Additional information for antimicrobial drug resistance and virulence gene profiles of *Salmonella enterica* serovar Enteritidis lineages in South Korea.
