# Supplementary material for: Emergence of Distinct Salmonella enterica Serovar Enteritidis Lineage since 2020, South Korea
Source: Emerg Infect Dis. 2025 Jul;31(7):1386–93. doi: 10.3201/eid3107.250043 (PMC12205472; doi:10.3201/eid3107.250043)
Supplement: Appendix 1 — Additional information for emergence of distinct Salmonella enterica serovar Enteritidis lineage since 2020, South Korea. [file 25-0043-Techapp-s1.pdf]

Article DOI: https://doi.org/10.3201/eid3107.250043
EID cannot ensure accessibility for supplementary materials supplied by authors. Readers who have difficulty accessing supplementary content should contact the authors for assistance.

Emergence of Distinct *Salmonella enterica* Serovar Enteritidis Lineage since 2020, South Korea

Appendix 1

Appendix 1 Table 1. Information on the 38 *Salmonella* Enteritidis isolates sequenced in this study*

Identification	Year	Collection date	Province	Source	Type†	Outbreak no.‡	Causative Foods	Bioproject no.	Reference	PFGE type	Korea clade	Global clade
23-GG-13–4945	2013	2013/11/06	Gyonggi	Human	Sporadic	NA	Unknown	PRJNA1150652	KDCA	SEGX01.049	Korea III	Global III
24-BS-14–3792	2014	2014/08/20	Busan	Human	Sporadic	NA	Unknown	PRJNA1150652	KDCA	SEGX01.049	Korea III	Global III
25-DJ-14–5950	2014	2014/10/14	Daejeon	Human	Sporadic	NA	Unknown	PRJNA1150652	KDCA	SEGX01.049	Korea V	Global I
01-DG-21–59	2020	2021/01/11	Daegu	Human	Outbreak	1	Braised burdock	PRJNA1150652	KDCA	SEGX01.049	Korea III	Global II
02-DG-21–69	2020	2021/01/11	Daegu	Human	Outbreak	1	Braised burdock	PRJNA1150652	KDCA	SEGX01.049	Korea III	Global II
03-DG-21–58	2020	2021/01/11	Daegu	Environment	Outbreak	1	Braised burdock	PRJNA1150652	KDCA	SEGX01.049	Korea III	Global II
04-CN-21–1530	2021	2021/06/29	Chungnam	Human	Outbreak	1	Braised burdock	PRJNA1150652	KDCA	SEGX01.049	Korea III	Global II
05-GG-21–1817	2021	2021/08/09	Gyonggi	Human	Outbreak	2	Egg in kimbap	PRJNA1150652	KDCA	SEGX01.049	Korea III	Global II
06-GG-21–1819	2021	2021/08/09	Gyonggi	Human	Outbreak	2	Egg in kimbap	PRJNA1150652	KDCA	SEGX01.049	Korea III	Global II
07-GG-21–1821	2021	2021/08/09	Gyonggi	Human	Outbreak	2	Egg in kimbap	PRJNA1150652	KDCA	SEGX01.049	Korea III	Global II
08-GG-21–1824	2021	2021/08/09	Gyonggi	Human	Outbreak	2	Egg in kimbap	PRJNA1150652	KDCA	SEGX01.049	Korea III	Global II
09-GG-21–1830	2021	2021/08/09	Gyonggi	Environment	Outbreak	2	Egg in kimbap	PRJNA1150652	KDCA	SEGX01.049	Korea III	Global II
10-GG-21–1831	2021	2021/08/09	Gyonggi	Environment	Outbreak	2	Egg in kimbap	PRJNA1150652	KDCA	SEGX01.049	Korea III	Global II
11-GG-21–1832	2021	2021/08/09	Gyonggi	Environment	Outbreak	2	Egg in kimbap	PRJNA1150652	KDCA	SEGX01.049	Korea III	Global II
12-GG-21–1833	2021	2021/08/09	Gyonggi	Environment	Outbreak	2	Egg in kimbap	PRJNA1150652	KDCA	SEGX01.049	Korea III	Global II
13-GN-21–2154	2021	2021/09/13	Gyeongnam	Human	Outbreak	3	Unknown	PRJNA1150652	KDCA	SEGX01.049	Korea III	Global II

Identification	Year	Collection date	Province	Source	Type†	Outbreak no.‡	Causative Foods	Bioproject no.	Reference	PFGE type	Korea clade	Global clade
17-GN-21–2201	2021	2021/10/07	Gyeongnam	Human	Outbreak	4	Sandwich	PRJNA1150652	KDCA	SEGX01.049	Korea III	Global II
18-GN-21–2229	2021	2021/10/07	Gyeongnam	Food	Outbreak	4	Sandwich	PRJNA1150652	KDCA	SEGX01.049	Korea III	Global II
19-JB-21–3443	2021	2021/11/18	Jeonbok	Human	Outbreak	5	Unknown	PRJNA1150652	KDCA	SEGX01.049	Korea III	Global II
20-JB-21–3450	2021	2021/11/18	Jeonbok	Human	Outbreak	5	Unknown	PRJNA1150652	KDCA	SEGX01.049	Korea III	Global II
21-GJ_S21	2022	2022/02/07	Gwangju	Environment	Outbreak	6	Egg in pan-fried, battered pollack fillet and shrimp	PRJNA1150652	KDCA	SEGX01.049	Korea III	Global II
22-GJ_S22	2022	2022/02/07	Gwangju	Human	Outbreak	6	Egg in pan-fried, battered pollack fillet and shrimp	PRJNA1150652	KDCA	SEGX01.049	Korea III	Global II
26-CN-BR	2022	2022/02/17	Chungnam	Human	Outbreak	7	Unknown	PRJNA1150652	KDCA	SEGX01.049	Korea III	Global II
27-CN-GJ	2022	2022/02/17	Chungnam	Human	Outbreak	7	Unknown	PRJNA1150652	KDCA	SEGX01.049	Korea III	Global II
23PH-SE1	2023	2023/11/17	Gyeongbok	Human	Outbreak	12	Egg in Soboro donburi	PRJNA1150652	KDCA	SEGX01.049	Korea III	Global II
23PH-SE10	2023	2023/11/17	Gyeongbok	Human	Outbreak	12	Egg in Soboro donburi	PRJNA1150652	KDCA	SEGX01.049	Korea III	Global II
23PH-SE11	2023	2023/11/17	Gyeongbok	Human	Outbreak	12	Egg in Soboro donburi	PRJNA1150652	KDCA	SEGX01.049	Korea III	Global II
23PH-SE12	2023	2023/11/17	Gyeongbok	Human	Outbreak	12	Egg in Soboro donburi	PRJNA1150652	KDCA	SEGX01.049	Korea III	Global II
23PH-SE13	2023	2023/11/17	Gyeongbok	Human	Outbreak	12	Egg in Soboro donburi	PRJNA1150652	KDCA	SEGX01.049	Korea III	Global II
23PH-SE14F	2023	2023/11/17	Gyeongbok	Human	Outbreak	12	Egg in Soboro donburi	PRJNA1150652	KDCA	SEGX01.049	Korea III	Global II
23PH-SE2	2023	2023/11/20	Gyeongbok	Human	Outbreak	12	Egg in Soboro donburi	PRJNA1150652	KDCA	SEGX01.049	Korea III	Global II
23PH-SE3	2023	2023/11/20	Gyeongbok	Human	Outbreak	12	Egg in Soboro donburi	PRJNA1150652	KDCA	SEGX01.049	Korea III	Global II
23PH-SE4	2023	2023/11/20	Gyeongbok	Human	Outbreak	12	Egg in Soboro donburi	PRJNA1150652	KDCA	SEGX01.049	Korea III	Global II
23PH-SE5	2023	2023/11/20	Gyeongbok	Human	Outbreak	12	Egg in Soboro donburi	PRJNA1150652	KDCA	SEGX01.049	Korea III	Global II
23PH-SE6	2023	2023/11/20	Gyeongbok	Human	Outbreak	12	Egg in Soboro donburi	PRJNA1150652	KDCA	SEGX01.049	Korea III	Global II
23PH-SE7	2023	2023/11/20	Gyeongbok	Human	Outbreak	12	Egg in Soboro donburi	PRJNA1150652	KDCA	SEGX01.049	Korea III	Global II
23PH-SE8	2023	2023/11/20	Gyeongbok	Human	Outbreak	12	Egg in Soboro donburi	PRJNA1150652	KDCA	SEGX01.049	Korea III	Global II
23PH-SE9	2023	2023/11/20	Gyeongbok	Human	Outbreak	12	Egg in Soboro donburi	PRJNA1150652	KDCA	SEGX01.049	Korea III	Global II

*KDCA, Korea Disease Control and Prevention Agency; NA, not applicable; PFGE, pulsed-field gel electrophoresis.
†Sporadic or outbreak.
‡Outbreak number according to Appendix 1 Table 4.

Appendix 1 Table 2. Information on the 316 *Salmonella* Enteritidis sequences used for phylogenetic analysis in this study*

Identification no.	Year	Country	Source	Reference	Bioproject or biosample no.
1_ChickenFarm	2022	South Korea	Chicken farm	NCBI	PRJNA1152726
190531_6	2019	South Korea	Poultry	NCBI	SAMN23139097
190604_4	2019	South Korea	Food	NCBI	SAMN23139099
190610_1	2019	South Korea	Poultry	NCBI	SAMN15870315
190617_3	2019	South Korea	Food	NCBI	SAMN23139100
190617_4	2019	South Korea	Food	NCBI	SAMN23139101
190704_2	2019	South Korea	Environment	NCBI	SAMN15870316
190729_4	2019	South Korea	Food	NCBI	SAMN15870317
190729_8	2019	South Korea	Food	NCBI	SAMN15870318
190807_1	2019	South Korea	Poultry	NCBI	SAMN15870319
190819_2	2019	South Korea	Food	NCBI	SAMN15870320
190821_1	2019	South Korea	Poultry	NCBI	SAMN15870321
191002_3	2019	South Korea	Poultry	NCBI	SAMN23139118
191108_1	2019	South Korea	Poultry	NCBI	SAMN23139119
2_ChickenFarm	2022	South Korea	Chicken farm	NCBI	PRJNA1152726
200320_2	2020	South Korea	Food	NCBI	SAMN23139120
200511_4	2020	South Korea	Food	NCBI	SAMN23139121
3_ChickenFarm	2022	South Korea	Chicken farm	NCBI	PRJNA1152726
4_ChickenFarm	2022	South Korea	Chicken farm	NCBI	PRJNA1152726
5_ChickenFarm	2022	South Korea	Chicken farm	NCBI	PRJNA1152726
F12–301	2012	South Korea	Environment	NCBI	SAMN23139123
F12–349	2012	South Korea	Environment	NCBI	SAMN23139125
F12–398	2012	South Korea	Environment	NCBI	SAMN23139126
F17–358	2017	South Korea	Environment	NCBI	SAMN23139127
F17–86	2017	South Korea	Poultry	NCBI	SAMN23139128
F17–91	2017	South Korea	Environment	NCBI	SAMN23139129
FDA449686_2–3	2007	South Korea	Aquatic animal	NCBI	SAMN02846029
FORC_007	2014	South Korea	Aquatic animal	NCBI	SAMN03140319
FORC_019	2014	South Korea	Human	NCBI	SAMN03896006
FORC_051	2016	South Korea	Human	NCBI	SAMN05756207
FORC_052	2014	South Korea	Environment	NCBI	SAMN05363482
FORC_056	2015	South Korea	Aquatic animal	NCBI	SAMN05631013
FORC_074	2002	South Korea	Human	NCBI	SAMN07604329
FORC_075	2017	South Korea	Human	NCBI	SAMN07580859
FORC50	2016	South Korea	Human	NCBI	SAMN06161238
K12SE001	N/A	South Korea	Poultry	NCBI	SAMN12586650
KR1	2011	South Korea	Retail chicken meat	NCBI	SRR11515115
KR10	2011	South Korea	Retail chicken meat	NCBI	SRR11515026
KR100	2012	South Korea	Chicken farm truck	NCBI	SRR11515025
KR101	2012	South Korea	Chicken farm truck	NCBI	SRR11515024
KR102	2012	South Korea	Chicken farm truck	NCBI	SRR11515023
KR103	2012	South Korea	Chicken farm truck	NCBI	SRR11515022
KR104	2012	South Korea	Chicken farm truck	NCBI	SRR11515021
KR105	2012	South Korea	Chicken farm truck	NCBI	SRR11515020
KR11	2011	South Korea	Retail chicken meat	NCBI	SRR11515113
KR12	2011	South Korea	Retail chicken meat	NCBI	SRR11515112
KR13	2011	South Korea	Retail chicken meat	NCBI	SRR11515111
KR14	2011	South Korea	Retail chicken meat	NCBI	SRR11515110
KR15	2011	South Korea	Retail chicken meat	NCBI	SRR11515109
KR16	2011	South Korea	Retail chicken meat	NCBI	SRR11515108
KR17	2011	South Korea	Retail chicken meat	NCBI	SRR11515107
KR18	2011	South Korea	Retail chicken meat	NCBI	SRR11515106
KR19	2011	South Korea	Retail chicken meat	NCBI	SRR11515105
KR2	2011	South Korea	Retail chicken meat	NCBI	SRR11515114
KR20	2011	South Korea	Retail chicken meat	NCBI	SRR11515104
KR21	2011	South Korea	Retail chicken meat	NCBI	SRR11515102
KR22	2011	South Korea	Retail chicken meat	NCBI	SRR11515101
KR23	2011	South Korea	Retail chicken meat	NCBI	SRR11515100
KR24	2011	South Korea	Retail chicken meat	NCBI	SRR11515099
KR25	2011	South Korea	Retail chicken meat	NCBI	SRR11515098
KR26	2011	South Korea	Retail chicken meat	NCBI	SRR11515097
KR27	2011	South Korea	Retail chicken meat	NCBI	SRR11515096
KR28	2011	South Korea	Retail chicken meat	NCBI	SRR11515095
KR29	2011	South Korea	Retail chicken meat	NCBI	SRR11515094
KR3	2011	South Korea	Retail chicken meat	NCBI	SRR11515103
KR30	2011	South Korea	Poultry slaughterhouse	NCBI	SRR11515093
KR31	2011	South Korea	Poultry slaughterhouse	NCBI	SRR11515091
KR32	2011	South Korea	Poultry slaughterhouse	NCBI	SRR11515090

Identification no.	Year	Country	Source	Reference	Bioproject or biosample no.
KR33	2011	South Korea	Poultry slaughterhouse	NCBI	SRR11515089
KR34	2011	South Korea	Poultry slaughterhouse	NCBI	SRR11515088
KR36	2011	South Korea	Poultry slaughterhouse	NCBI	SRR11515087
KR39	2011	South Korea	Livestock	NCBI	SRR11515086
KR4	2010	South Korea	Livestock	NCBI	SRR11515092
KR40	2011	South Korea	Poultry slaughterhouse	NCBI	SRR11515085
KR41	2011	South Korea	Poultry slaughterhouse	NCBI	SRR11515084
KR43	2011	South Korea	Wild Animal	NCBI	SRR11515083
KR44	2010	South Korea	Poultry slaughterhouse	NCBI	SRR11515082
KR45	2010	South Korea	Poultry slaughterhouse	NCBI	SRR11515080
KR46	2010	South Korea	Poultry slaughterhouse	NCBI	SRR11515079
KR47	2010	South Korea	Poultry slaughterhouse	NCBI	SRR11515078
KR48	2010	South Korea	Poultry slaughterhouse	NCBI	SRR11515077
KR49	2010	South Korea	Poultry slaughterhouse	NCBI	SRR11515076
KR5	2011	South Korea	Retail chicken meat	NCBI	SRR11515081
KR50	2010	South Korea	Livestock	NCBI	SRR11515075
KR51	2010	South Korea	Chicken farm truck	NCBI	SRR11515073
KR53	2011	South Korea	Livestock	NCBI	SRR11515073
KR54	2011	South Korea	Chicken farm	NCBI	SRR11515072
KR55	2011	South Korea	Chicken farm	NCBI	SRR11515071
KR56	2011	South Korea	Chicken farm	NCBI	SRR11515069
KR57	2017	South Korea	Chicken farm	NCBI	SRR11515068
KR58	2017	South Korea	Chicken farm	NCBI	SRR11515067
KR59	2017	South Korea	Chicken farm	NCBI	SRR11515066
KR6	2011	South Korea	Retail chicken meat	NCBI	SRR11515070
KR60	2017	South Korea	Chicken farm	NCBI	SRR11515065
KR61	2017	South Korea	Chicken farm	NCBI	SRR11515064
KR62	2017	South Korea	Chicken farm	NCBI	SRR11515063
KR63	2017	South Korea	Chicken farm	NCBI	SRR11515062
KR64	2017	South Korea	Chicken farm	NCBI	SRR11515061
KR65	2017	South Korea	Chicken farm	NCBI	SRR11515060
KR66	2017	South Korea	Chicken farm	NCBI	SRR11515058
KR67	2017	South Korea	Chicken farm	NCBI	SRR11515057
KR68	2017	South Korea	Chicken farm	NCBI	SRR11515056
KR69	2017	South Korea	Chicken farm	NCBI	SRR11515055
KR7	2011	South Korea	Retail chicken meat	NCBI	SRR11515059
KR70	2017	South Korea	Chicken farm	NCBI	SRR11515054
KR71	2017	South Korea	Chicken farm	NCBI	SRR11515053
KR73	2017	South Korea	Chicken farm	NCBI	SRR11515052
KR74	2017	South Korea	Chicken farm	NCBI	SRR11515051
KR75	2017	South Korea	Chicken farm	NCBI	SRR11515050
KR76	2017	South Korea	Chicken farm	NCBI	SRR11515049
KR77	2017	South Korea	Chicken farm	NCBI	SRR11515047
KR78	2017	South Korea	Chicken farm	NCBI	SRR11515046
KR79	2017	South Korea	Chicken farm	NCBI	SRR11515045
KR8	2011	South Korea	Retail chicken meat	NCBI	SRR11515048
KR81	2013	South Korea	Chicken farm	NCBI	SRR11515044
KR82	2013	South Korea	Chicken farm	NCBI	SRR11515043
KR83	2014	South Korea	Chicken farm	NCBI	SRR11515042
KR84	2014	South Korea	Duck farm	NCBI	SRR11515041
KR85	2014	South Korea	Chicken farm	NCBI	SRR11515040
KR86	2015	South Korea	Chicken farm	NCBI	SRR11515039
KR87	2015	South Korea	Chicken farm	NCBI	SRR11515038
KR88	2016	South Korea	Chicken farm	NCBI	SRR11515036
KR9	2011	South Korea	Retail chicken meat	NCBI	SRR11515037
KR90	2016	South Korea	Duck farm	NCBI	SRR11515035
KR91	2017	South Korea	Chicken farm	NCBI	SRR11515034
KR92	2017	South Korea	Chicken farm	NCBI	SRR11515033
KR93	2017	South Korea	Chicken farm	NCBI	SRR11515032
KR94	2017	South Korea	Chicken farm	NCBI	SRR11515031
KR95	2017	South Korea	Chicken farm	NCBI	SRR11515030
KR96	2012	South Korea	Poultry slaughterhouse	NCBI	SRR11515029
KR97	2012	South Korea	Poultry slaughterhouse	NCBI	SRR11515028
KR99	2012	South Korea	Chicken farm truck	NCBI	SRR11515027
KUFSE-CM123	2013	South Korea	Food	NCBI	SAMN14167843
KUFSE-CM132	2013	South Korea	Food	NCBI	SAMN14167844
KUFSE-CM149	2013	South Korea	Food	NCBI	SAMN14167846
KUFSE-CM160	2014	South Korea	Food	NCBI	SAMN14167847
KUFSE-CM161	2014	South Korea	Food	NCBI	SAMN14167848

Identification no.	Year	Country	Source	Reference	Bioproject or biosample no.
KUFSE-CM22	2012	South Korea	Food	NCBI	SAMN14167839
KUFSE-CM34	2012	South Korea	Food	NCBI	SAMN14167841
MFDS1004839	2014	South Korea	Food	NCBI	SAMN08323710
MFDS1018147(CP110220)	2020	South Korea	Food	NCBI	SAMN31231980
NCCP_16206	2011	South Korea	Human	NCBI	SAMN12340930
SAL_AB8575AA_AS	2018	United Kingdom	Human	Enterobase	SRR7172572
SAL_DD0468AA_AS	2022	United Kingdom	Human	Enterobase	SRR25879005
SAL_DD0936AA_AS	2022	United Kingdom	Human	Enterobase	SRR25872738
SAL_DD1013AA_AS	2022	United Kingdom	Human	Enterobase	SRR25872644
SAL_DD1288AA_AS	2022	United Kingdom	Human	Enterobase	SRR25872313
SAL_DD1337AA_AS	2022	United Kingdom	Human	Enterobase	SRR25872654
SAL_EB0226AA_AS	2018	United Kingdom	Human	Enterobase	SRR7828297
SAL_EB0310AA_AS	2018	United Kingdom	Human	Enterobase	SRR7828445
SAL_EB0348AA_AS	2018	United Kingdom	Human	Enterobase	SRR7828483
SAL_EB0699AA_AS	2018	United Kingdom	Human	Enterobase	SRR7841633
SAL_EB5405AA_AS	2018	United Kingdom	Human	Enterobase	SRR7892238
SAL_EB8869AA_AS	2018	United Kingdom	Human	Enterobase	SRR7998226
SAL_EC2476AA_AS	2020	United Kingdom	Human	Enterobase	SRR11410341
SAL_EC2535AA_AS	2020	United Kingdom	Human	Enterobase	SRR11410500
SAL_FB0875AA_AS	2018	United Kingdom	Human	Enterobase	SRR8087123
SAL_FB4774AA_AS	2018	United Kingdom	Human	Enterobase	SRR8131547
SAL_FB7348AA_AS	2018	United Kingdom	Human	Enterobase	SRR8182586
SAL_FB7389AA_AS	2018	United Kingdom	Human	Enterobase	SRR8183176
SAL_GB1159AA_AS	2018	United Kingdom	Human	Enterobase	SRR8201767
SAL_GB1239AA_AS	2018	United Kingdom	Human	Enterobase	SRR8201852
SAL_GB5321AA_AS	2018	United Kingdom	Human	Enterobase	SRR8249729
SAL_HB5640AA_AS	2018	United Kingdom	Human	Enterobase	SRR8350282
SAL_HC3884AA_AS	2020	United Kingdom	Human	Enterobase	SRR11607190
SAL_IB5605AA_AS	2018	United Kingdom	Human	Enterobase	SRR8490725
SAL_IB7246AA_AS	2018	United Kingdom	Human	Enterobase	SRR8503801
SAL_IB7643AA_AS	2018	United Kingdom	Human	Enterobase	SRR8509387
SAL_IC3482AA_AS	2020	United Kingdom	Human	Enterobase	SRR12177365
SAL_IC9527AA_AS	2020	United Kingdom	Human	Enterobase	SRR12430844
SAL_JB9475AA_AS	2019	United Kingdom	Human	Enterobase	SRR8648175
SAL_JC5401AA_AS	2020	United Kingdom	Human	Enterobase	SRR12574346
SAL_JC6905AA_AS	2020	United Kingdom	Human	Enterobase	SRR12594930
SAL_KB3398AA_AS	2019	United Kingdom	Human	Enterobase	SRR8706107
SAL_KC0777AA_AS	2020	United Kingdom	Human	Enterobase	SRR12645911
SAL_KC0826AA_AS	2020	United Kingdom	Human	Enterobase	SRR12645979
SAL_KC2021AA_AS	2020	United Kingdom	Human	Enterobase	SRR12676804
SAL_KC7347AA_AS	2020	United Kingdom	Human	Enterobase	SRR12770212
SAL_LC3709AA_AS	2020	United Kingdom	Human	Enterobase	SRR12956729
SAL_LC3827AA_AS	2020	United Kingdom	Human	Enterobase	SRR12959014
SAL_NB1183AA_AS	2019	United Kingdom	Human	Enterobase	SRR9335610
SAL_NB7091AA_AS	2019	United Kingdom	Human	Enterobase	SRR9649499
SAL_NC6353AA_AS	2021	United Kingdom	Human	Enterobase	SRR13779303
SAL_PB5495AA_AS	2019	United Kingdom	Human	Enterobase	SRR9989248
SAL_PB6059AA_AS	2019	United Kingdom	Human	Enterobase	SRR10006887
SAL_PB8568AA_AS	2019	United Kingdom	Human	Enterobase	SRR10018245
SAL_PB8614AA_AS	2019	United Kingdom	Human	Enterobase	SRR10018342
SAL_PB9134AA_AS	2019	United Kingdom	Human	Enterobase	SRR10019374
SAL_QB2233AA_AS	2019	United Kingdom	Human	Enterobase	SRR10054366
SAL_QB3066AA_AS	2019	United Kingdom	Human	Enterobase	SRR10062992
SAL_RB8022AA_AS	2019	United Kingdom	Human	Enterobase	SRR10198872
SAL_RB9660AA_AS	2019	United Kingdom	Human	Enterobase	SRR10222104
SAL_RB9673AA_AS	2019	United Kingdom	Human	Enterobase	SRR10222117
SAL_RC1563AA_AS	2018	United Kingdom	ND	Enterobase	SRR15366233
SAL_RC7598AA_AS	2021	United Kingdom	Human	Enterobase	SRR16054251
SAL_SB0310AA_AS	2019	United Kingdom	Human	Enterobase	SRR10229761
SAL_SB0955AA_AS	2019	United Kingdom	Human	Enterobase	SRR10232144
SAL_SB1662AA_AS	2019	United Kingdom	Human	Enterobase	SRR10235809
SAL_SC1704AA_AS	2021	United Kingdom	Human	Enterobase	SRR16291929
SAL_SC8599AA_AS	2021	United Kingdom	Human	Enterobase	SRR17038761
SAL_TB5660AA_AS	2019	United Kingdom	Human	Enterobase	SRR10386721
SAL_TC4386AA_AS	2022	United Kingdom	Human	Enterobase	SRR17900636
SAL_TC7849AA_AS	2021	South Africa	Human	Enterobase	ERR9420185
SAL_UC2533AA_AS	2022	United Kingdom	Human	Enterobase	SRR19136134
SAL_UC2614AA_AS	2022	United Kingdom	Human	Enterobase	SRR19100831
SAL_UC3100AA_AS	2022	United Kingdom	Human	Enterobase	SRR19139585

Identification no.	Year	Country	Source	Reference	Bioproject or biosample no.
SAL_UC5098AA_AS	2022	United Kingdom	Human	Enterobase	SRR19364516
SAL_UC5400AA_AS	2022	United Kingdom	Human	Enterobase	SRR19325695
SAL_VB8873AA_AS	2019	United Kingdom	Human	Enterobase	SRR10519600
SAL_VB8975AA_AS	2019	United Kingdom	Human	Enterobase	SRR10542570
SAL_VC0275AA_AS	2022	United Kingdom	Human	Enterobase	SRR19588452
SAL_VC2869AA_AS	2022	United Kingdom	Human	Enterobase	SRR19846887
SAL_VC5140AA_AS	2022	United Kingdom	Human	Enterobase	SRR20053626
SAL_VC5187AA_AS	2022	United Kingdom	Human	Enterobase	SRR20053260
SAL_VC5629AA_AS	2022	United Kingdom	Human	Enterobase	SRR19973695
SAL_VC6111AA_AS	2022	United Kingdom	Human	Enterobase	SRR20230890
SAL_WB3333AA_AS	2019	United Kingdom	Human	Enterobase	SRR10604685
SAL_WC2230AA_AS	2022	United Kingdom	Human	Enterobase	SRR21142569
SAL_WC2261AA_AS	2022	United States	Human	Enterobase	SRR21160206
SAL_WC5739AA_AS	2022	United Kingdom	Human	Enterobase	SRR21424469
SAL_WC8114AA_AS	2022	United Kingdom	Human	Enterobase	SRR21594912
SAL_WC8741AA_AS	2022	United Kingdom	Human	Enterobase	SRR21629163
SAL_WC9106AA_AS	2022	United Kingdom	Human	Enterobase	SRR21608997
SAL_XC4763AA_AS	2022	United Kingdom	Human	Enterobase	SRR21910798
SAL_XC6393AA_AS	2022	United Kingdom	Human	Enterobase	SRR21424466
SAL_XC6397AA_AS	2022	United Kingdom	Human	Enterobase	SRR21424452
SAL_YC1440AA_AS	2022	United Kingdom	Human	Enterobase	SRR22221227
SAL_YC1448AA_AS	2022	United Kingdom	Human	Enterobase	SRR22221210
SAL_YC3817AA_AS	2022	United Kingdom	Human	Enterobase	SRR22257345
SAL_YC4393AA_AS	2022	United Kingdom	Human	Enterobase	SRR21205287
SAL_YC4516AA_AS	2022	United Kingdom	Human	Enterobase	SRR21284441
SAL_YC8448AA_AS	2022	United Kingdom	Human	Enterobase	SRR22691114
SAL_ZC4770AA_AS	2022	United Kingdom	Human	Enterobase	SRR22990945
SEGX001–15GG-976	2015	South Korea	Human	NCBI	PRJNA1155254
SEGX001–18IC-628	2018	South Korea	Human	NCBI	PRJNA1155254
SEGX001–21GJ-1621	2021	South Korea	Human	NCBI	PRJNA1155254
SEGX001–21GW-1463	2021	South Korea	Human	NCBI	PRJNA1155254
SEGX002–15GB-3715	2015	South Korea	Human	NCBI	PRJNA1155254
SEGX002–17JB-2242	2017	South Korea	Human	NCBI	PRJNA1155254
SEGX003–09DJ-1671	2009	South Korea	Human	NCBI	PRJNA1155254
SEGX003–09GB-891	2009	South Korea	Human	NCBI	PRJNA1155254
SEGX003–09JJ-1591	2009	South Korea	Human	NCBI	PRJNA1155254
SEGX003–10DJ-136	2010	South Korea	Human	NCBI	PRJNA1155254
SEGX003–10DJ-1498	2010	South Korea	Human	NCBI	PRJNA1155254
SEGX003–11DG-406	2011	South Korea	Human	NCBI	PRJNA1155254
SEGX003–11SE-220	2011	South Korea	Human	NCBI	PRJNA1155254
SEGX003–12GJ-377	2012	South Korea	Human	NCBI	PRJNA1155254
SEGX003–13GN-1551	2013	South Korea	Human	NCBI	PRJNA1155254
SEGX003–14GB-3346	2014	South Korea	Human	NCBI	PRJNA1155254
SEGX003–14GJ-4961	2014	South Korea	Human	NCBI	PRJNA1155254
SEGX003–14GN-1879	2014	South Korea	Human	NCBI	PRJNA1155254
SEGX003–14IC-4775	2014	South Korea	Human	NCBI	PRJNA1155254
SEGX003–14JJ-1334	2014	South Korea	Human	NCBI	PRJNA1155254
SEGX003–14SE-2940	2014	South Korea	Human	NCBI	PRJNA1155254
SEGX003–15–843	2015	South Korea	Human	NCBI	PRJNA1155254
SEGX003–15GJ-65	2015	South Korea	Human	NCBI	PRJNA1155254
SEGX003–15IC-1480	2015	South Korea	Human	NCBI	PRJNA1155254
SEGX003–17JJ-456	2017	South Korea	Human	NCBI	PRJNA1155254
SEGX003–18JJ-2222	2018	South Korea	Human	NCBI	PRJNA1155254
SEGX004–15–1313	2015	South Korea	Human	NCBI	PRJNA1155254
SEGX004–18GN-2166	2018	South Korea	Human	NCBI	PRJNA1155254
SEGX005–05CN-5187	2005	South Korea	Human	NCBI	PRJNA1155254
SEGX005–05GJ-1046	2005	South Korea	Human	NCBI	PRJNA1155254
SEGX007–15–665	2015	South Korea	Human	NCBI	PRJNA1155254
SEGX008–09JB-1298	2009	South Korea	Human	NCBI	PRJNA1155254
SEGX009–13GJ-4851	2013	South Korea	Human	NCBI	PRJNA1155254
SEGX009–14GJ-4243	2014	South Korea	Human	NCBI	PRJNA1155254
SEGX011–10IC-1064	2010	South Korea	Human	NCBI	PRJNA1155254
SEGX011–13GJ-1611	2013	South Korea	Human	NCBI	PRJNA1155254
SEGX011–14GJ-4237	2014	South Korea	Human	NCBI	PRJNA1155254
SEGX013–12BS-330	2012	South Korea	Human	NCBI	PRJNA1155254
SEGX016–14JN-3127	2014	South Korea	Human	NCBI	PRJNA1155254
SEGX016–15SE-229	2015	South Korea	Human	NCBI	PRJNA1155254
SEGX017–14GJ-4244	2014	South Korea	Human	NCBI	PRJNA1155254
SEGX018–09GN-1344	2009	South Korea	Human	NCBI	PRJNA1155254

Identification no.	Year	Country	Source	Reference	Bioproject or biosample no.
SEGX019–11DJ-442	2011	South Korea	Human	NCBI	PRJNA1155254
SEGX020–10DG-1129	2010	South Korea	Human	NCBI	PRJNA1155254
SEGX020–10DJ-133	2010	South Korea	Human	NCBI	PRJNA1155254
SEGX020–10DJ-2616	2010	South Korea	Human	NCBI	PRJNA1155254
SEGX020–10SE-1855	2010	South Korea	Human	NCBI	PRJNA1155254
SEGX020–11SE-55	2011	South Korea	Human	NCBI	PRJNA1155254
SEGX023–09DG-1506	2009	South Korea	Human	NCBI	PRJNA1155254
SEGX024–10DJ-2604	2010	South Korea	Human	NCBI	PRJNA1155254
SEGX024–10IC-1086	2010	South Korea	Human	NCBI	PRJNA1155254
SEGX025–11GN-2733	2011	South Korea	Human	NCBI	PRJNA1155254
SEGX025–14JN-4303	2014	South Korea	Human	NCBI	PRJNA1155254
SEGX026–10GN-1472	2010	South Korea	Human	NCBI	PRJNA1155254
SEGX026–15GGN-1679	2015	South Korea	Human	NCBI	PRJNA1155254
SEGX027–09CN-509	2009	South Korea	Human	NCBI	PRJNA1155254
SEGX027–09CN-510	2009	South Korea	Human	NCBI	PRJNA1155254
SEGX028–09GN-1351	2009	South Korea	Human	NCBI	PRJNA1155254
SEGX042–11JN-370	2011	South Korea	Human	NCBI	PRJNA1155254
SEGX042–12JB-2693	2012	South Korea	Human	NCBI	PRJNA1155254
SEGX042–13GB-271	2013	South Korea	Human	NCBI	PRJNA1155254
SEGX042–18BR-2687	2018	South Korea	Human	NCBI	PRJNA1155254
SEGX054–11IC-156	2011	South Korea	Human	NCBI	PRJNA1155254
SEGX056–10JN-2660	2010	South Korea	Human	NCBI	PRJNA1155254
SEGX078–12BR-1830	2012	South Korea	Human	NCBI	PRJNA1155254
SEGX089–18GW-2024	2018	South Korea	Human	NCBI	PRJNA1155254
SEGX089–20CN-1252	2020	South Korea	Human	NCBI	PRJNA1155254
SEGX089–21CN-2194	2021	South Korea	Human	NCBI	PRJNA1155254
SEGX089–21GGN-2104	2021	South Korea	Human	NCBI	PRJNA1155254
SEGX092–13GB-2648	2013	South Korea	Human	NCBI	PRJNA1155254
SEGX105–13GB-3543	2013	South Korea	Human	NCBI	PRJNA1155254
SEGX105–15GJ-1207	2015	South Korea	Human	NCBI	PRJNA1155254
SEGX131–18GJ-2943	2018	South Korea	Human	NCBI	PRJNA1155254
SEGX131–21GG-2185	2021	South Korea	Human	NCBI	PRJNA1155254
SEGX132–18BR-798	2018	South Korea	Human	NCBI	PRJNA1155254
SEGX132–18IC-1241	2018	South Korea	Human	NCBI	PRJNA1155254
SRI-A11	2011	South Korea	Food	NCBI	SAMN23139130
SRI-A14	2011	South Korea	Food	NCBI	SAMN23139131
SRI-A21	2011	South Korea	Food	NCBI	SAMN23139132
YUMC_B8891	2017	South Korea	Human	NCBI	SAMN15929509
YUMC_S147	2017	South Korea	Human	NCBI	SAMN15929510

NCBI, National Center for Biology Information.

Appendix 1 Table 3. Number of isolates and outbreaks of the 10 most prevalent PFGE patterns of the *Salmonella* Enteritidis isolates submitted to the Korea Centers for Diseases Control and Prevention Agency through the PulseNet Korea and EnterNet Korea systems during 2013–2024*

	Year												
PFGE types	2013	2014	2015	2016	2017	2018	2019	2020	2021	2022	2023	Aug-24	Total
No. (%) isolates													
SEGX01.049	1 (1.1)	2 (1.3)	NA	NA	NA	3 (3.2)	NA	11 (38.0)	110 (54.2)	32 (18.1)	60 (36.4)	165 (96.5)	384 (29.0)
SEGX01.089	2 (2.1)	2 (1.3)	NA	NA	NA	6 (6.4)	NA	17 (58.6)	49 (24.1)	32 (18.1)	69 (41.8)	NA	177 (13.4)
SEGX01.002	45 (47.4)	21 (13.5)	19 (19.8)	21 (43.8)	32 (41.0)	10 (10.7)	NA	NA	NA	NA	NA	NA	144 (10.9)
SEGX01.003	2 (2.1)	38 (24.4)	33 (34.4)	6 (12.5)	12 (15.4)	10 (10.7)	NA	NA	NA	NA	NA	NA	105 (7.9)
SEGX01.157	19 (20.0)	NA	NA	NA	NA	NA	NA	NA	NA	76 (42.3)	NA	NA	88 (6.7)
SEGX01.001	NA	16 (10.3)	24 (25)	1 (2.1)	5 (6.4)	5 (5.3)	3 (25)	NA	14 (6.9)		1 (0.6)	NA	76 (5.7)
SEGX01.156	4 (4.2)	NA	NA	NA	NA	NA	NA	NA	NA	18 (10.2)	14 (8.5)	6 (3.5)	42 (3.2)
SEGX01.007	NA	11 (7.1)	11 (11.5)	3 (6.25)	2 (2.6)	4 (4.3)	7 (58.3)	NA	NA	NA	NA	NA	38 (2.9)
SEGX01.025	NA	36 (23.1)	NA	NA	NA	1 (1.0)	NA	NA	NA	NA	NA	NA	37 (2.8)
SEGX01.004	1 (1.1)	NA	1 (1.0)	5 (10.4)	12 (15.4)	3 (3.2)	NA	NA	1 (0.5)	NA	NA	NA	23 (1.7)
SEGX01.132	NA	NA	NA	NA	NA	2 (2.1)	NA	NA	18 (8.9)	NA	NA	NA	20 (1.5)
Total	74 (77.9)	126 (80.8)	88 (91.7)	36 (75)	63 (80.8)	44 (46.8)	10 (83.3)	28 (100)	192 (94.6)	158 (89.3)	144 (87.3)	171 (100)	1,124 (84.9)
No. isolates submitted to PulseNet and EnterNet Korea system	95 (100)	156 (100)	96 (100)	48 (100)	78 (100)	94 (100)	12 (100)	29 (100)	203 (100)	177 (100)	165 (100)	171 (100)	1324 (100)
No. (%) outbreaks													
SEGX01.049	NA	NA	NA	NA	NA	NA	NA	1 (33.3)	3 (42.9)	4 (33.3)	4 (28.6)	11 (91.7)	23 (35.9)
SEGX01.089	NA	NA	NA	NA	NA	NA	NA	2 (66.7)	3 (42.9)	3 (25.0)	9 (64.3)	NA	17 (26.6)
SEGX01.002	NA	1 (50)	NA	2 (66.7)	2 (40)	NA	NA	NA	NA	NA	NA	NA	5 (7.8)
SEGX01.003	2 (100)	NA	1 (100)	1 (33.3)	2 (40)	2 (100)	NA	NA	NA	NA	NA	NA	8 (12.5)
SEGX01.157	NA	NA	NA	NA	NA	NA	NA	NA	NA	NA	NA	NA	0
SEGX01.001	NA	NA	NA	NA	NA	NA	NA	NA	NA	NA	NA	NA	0
SEGX01.156	NA	NA	NA	NA	NA	NA	NA	NA	NA	4 (33.3)	1 (7.1)	1 (8.3)	6 (9.4)
SEGX01.007	NA	NA	NA	NA	NA	NA	1 (100)	NA	NA	NA	NA	NA	1 (1.6)
SEGX01.025	NA	1 (50)	NA	NA	NA	NA	NA	NA	NA	NA	NA	NA	1 (1.6)
SEGX01.004	NA	NA	NA	NA	1 (20)	NA	NA	NA	NA	NA	NA	NA	1 (1.6)
SEGX01.132	NA	NA	NA	NA	NA	NA	NA	NA	NA	1 (8.3)	NA	NA	1 (1.6)
SEGX01.131	NA	NA	NA	NA	NA	NA	NA	NA	1 (14.3)	NA	NA	NA	1 (1.6)
Total	2 (100)	2 (100)	1 (100)	3 (100)	5 (100)	2 (100)	1 (100)	3 (100)	7 (100)	12 (100)	14 (100)	12 (100)	64 (100)

*NA, not applicable; PFGE, pulsed-field gel electrophoresis.
†Number of isolates/number of isolates submitted to the PulseNet Korea and EnterNet Korea system.

Appendix 1 Table 4. Information on the SEGX01.049 outbreaks in South Korea

Outbreak no.	Year	Region	No. cases	Causative food
1	2020	Daegu	11	Braised burdock
2	2021	Gyonggi	22	Egg in kimbap
3	2021	Gyeongnam	17	Unknown
4	2021	Gyeongnam	22	Egg in sandwich
5	2021	Jeonbok	15	Unknown
6	2022	Gwangju	5	Egg in pan-fried, battered pollack fillet and shrimp
7	2022	Chungnam	2	Unknown
8	2022	Gwangju	24	Unknown
9	2022	Chungnam	22	Egg in egg roll
10	2023	Gyonggi	26	Egg in kimbap
11	2023	Chungnam	2	Unknown
12	2023	Gyeongbok	32	Egg in Soboro donburi
13	2024	Busan	38	Egg in pan-fried, battered Meat
14	2024	Gyeongnam	15	Egg in pan-fried battered meat
15	2024	Busan	20	Egg in kimbap
16	2024	Ulsan	30	Unknown
17	2024	Busan	19	Egg in kimbap
18	2024	Busan	35	Egg in egg roll
19	2024	Seoul	33	Omurice
20	2024	Chungnam	5	Unknown
21	2024	Busan	6	Unknown
22	2024	Sejong	6	Unknown
23	2024	Daegu	3	Unknown

Appendix 1 Table 5. Results of Bayesian phylogeographic analysis of Global II clades

From	To	Bayes factor	Posterior probability
Other countries in Europe	United Kingdom	29353.79396	1
United Kingdom	United States	9782.423632	0.999666704
United Kingdom	Africa	1394.693491	0.997666926
United Kingdom	Korea	111.4144654	0.971558716
Other countries in Europe	Africa	7.321400104	0.691812021
Africa	United States	4.56701547	0.583379624
United States	Africa	4.285268236	0.567825797

Appendix 1 Table 6. Prophages of *Salmonella* Enteritidis isolates from our study (red font) and other isolates within Global IIa clades (blue font)

Strain	Global clade	Region	Region size, kb	most_common_phage_name
01_DG_21_59_korea	Global IIa	1	43.7	PHAGE_Salmon_RE_2010_NC_019488
01_DG_21_59_korea	Global IIa	2	25.7	PHAGE_Shigel_POCJ13_NC_025434
01_DG_21_59_korea	Global IIa	3	31.1	PHAGE_Phage_Gifsy_2_NC_010393
01_DG_21_59_korea	Global IIa	4	9.9	PHAGE_Phage_Gifsy_2_NC_010393
02_DG_21_69_korea	Global IIa	1	43.7	PHAGE_Salmon_RE_2010_NC_019488
02_DG_21_69_korea	Global IIa	2	9.8	PHAGE_Phage_Gifsy_2_NC_010393
02_DG_21_69_korea	Global IIa	3	31.1	PHAGE_Phage_Gifsy_2_NC_010393
02_DG_21_69_korea	Global IIa	4	25.7	PHAGE_Shigel_POCJ13_NC_025434
03_DG_21_58_korea	Global IIa	1	43.7	PHAGE_Salmon_RE_2010_NC_019488
03_DG_21_58_korea	Global IIa	2	25.7	PHAGE_Shigel_Stx_NC_029120
03_DG_21_58_korea	Global IIa	3	31.1	PHAGE_Phage_Gifsy_2_NC_010393
03_DG_21_58_korea	Global IIa	4	9.9	PHAGE_Phage_Gifsy_2_NC_010393
04_CN_21_1530_korea	Global IIa	1	9.8	PHAGE_Phage_Gifsy_2_NC_010393
04_CN_21_1530_korea	Global IIa	2	31.1	PHAGE_Phage_Gifsy_2_NC_010393
04_CN_21_1530_korea	Global IIa	3	25.7	PHAGE_Shigel_POCJ13_NC_025434
04_CN_21_1530_korea	Global IIa	4	43.7	PHAGE_Salmon_RE_2010_NC_019488
05_GG_21_1817_korea	Global IIa	1	25.7	PHAGE_Shigel_Stx_NC_029120
05_GG_21_1817_korea	Global IIa	2	31.1	PHAGE_Phage_Gifsy_2_NC_010393
05_GG_21_1817_korea	Global IIa	3	9.9	PHAGE_Phage_Gifsy_2_NC_010393
05_GG_21_1817_korea	Global IIa	4	43.7	PHAGE_Salmon_RE_2010_NC_019488
06_GG_21_1819_korea	Global IIa	1	25.7	PHAGE_Shigel_Stx_NC_029120
06_GG_21_1819_korea	Global IIa	2	31.1	PHAGE_Phage_Gifsy_2_NC_010393
06_GG_21_1819_korea	Global IIa	3	43.7	PHAGE_Salmon_RE_2010_NC_019488
06_GG_21_1819_korea	Global IIa	4	9.9	PHAGE_Phage_Gifsy_2_NC_010393
07_GG_21_1821_korea	Global IIa	1	43.7	PHAGE_Salmon_RE_2010_NC_019488
07_GG_21_1821_korea	Global IIa	2	31.1	PHAGE_Phage_Gifsy_2_NC_010393
07_GG_21_1821_korea	Global IIa	3	25.7	PHAGE_Shigel_Stx_NC_029120
07_GG_21_1821_korea	Global IIa	4	9.8	PHAGE_Phage_Gifsy_2_NC_010393
08_GG_21_1824_korea	Global IIa	1	43.7	PHAGE_Salmon_RE_2010_NC_019488
08_GG_21_1824_korea	Global IIa	2	9.8	PHAGE_Phage_Gifsy_2_NC_010393
08_GG_21_1824_korea	Global IIa	3	31.1	PHAGE_Phage_Gifsy_2_NC_010393
08_GG_21_1824_korea	Global IIa	4	25.7	PHAGE_Shigel_POCJ13_NC_025434
09_GG_21_1830_korea	Global IIa	1	43.7	PHAGE_Salmon_RE_2010_NC_019488
09_GG_21_1830_korea	Global IIa	2	25.7	PHAGE_Shigel_Stx_NC_029120
09_GG_21_1830_korea	Global IIa	3	31.1	PHAGE_Phage_Gifsy_2_NC_010393
09_GG_21_1830_korea	Global IIa	4	9.9	PHAGE_Phage_Gifsy_2_NC_010393
10_GG_21_1831_korea	Global IIa	1	25.7	PHAGE_Shigel_POCJ13_NC_025434
10_GG_21_1831_korea	Global IIa	2	31.1	PHAGE_Phage_Gifsy_2_NC_010393
10_GG_21_1831_korea	Global IIa	3	9.9	PHAGE_Phage_Gifsy_2_NC_010393
10_GG_21_1831_korea	Global IIa	4	43.7	PHAGE_Salmon_RE_2010_NC_019488
11_GG_21_1832_korea	Global IIa	1	9.8	PHAGE_Phage_Gifsy_2_NC_010393
11_GG_21_1832_korea	Global IIa	2	31.1	PHAGE_Phage_Gifsy_2_NC_010393
11_GG_21_1832_korea	Global IIa	3	25.7	PHAGE_Shigel_Stx_NC_029120
11_GG_21_1832_korea	Global IIa	4	43.7	PHAGE_Salmon_RE_2010_NC_019488
12_GG_21_1833_korea	Global IIa	1	43.7	PHAGE_Salmon_RE_2010_NC_019488
12_GG_21_1833_korea	Global IIa	2	25.7	PHAGE_Shigel_POCJ13_NC_025434
12_GG_21_1833_korea	Global IIa	3	31.1	PHAGE_Phage_Gifsy_2_NC_010393
12_GG_21_1833_korea	Global IIa	4	9.9	PHAGE_Phage_Gifsy_2_NC_010393

Strain	Global clade	Region	Region size, kb	most_common_phage_name
13_GN_21_2154_korea	Global IIa	1	9.8	PHAGE_Phage_Gifsy_2_NC_010393
13_GN_21_2154_korea	Global IIa	2	31.1	PHAGE_Phage_Gifsy_2_NC_010393
13_GN_21_2154_korea	Global IIa	3	25.7	PHAGE_Shigel_POCJ13_NC_025434
13_GN_21_2154_korea	Global IIa	4	43.7	PHAGE_Salmon_RE_2010_NC_019488
17_GN_21_2201_korea	Global IIa	1	25.7	PHAGE_Shigel_Stx_NC_029120
17_GN_21_2201_korea	Global IIa	2	31.1	PHAGE_Phage_Gifsy_2_NC_010393
17_GN_21_2201_korea	Global IIa	3	9.9	PHAGE_Phage_Gifsy_2_NC_010393
17_GN_21_2201_korea	Global IIa	4	43.7	PHAGE_Salmon_RE_2010_NC_019488
18_GN_21_2229_korea	Global IIa	1	25.7	PHAGE_Shigel_POCJ13_NC_025434
18_GN_21_2229_korea	Global IIa	2	31.1	PHAGE_Phage_Gifsy_2_NC_010393
18_GN_21_2229_korea	Global IIa	3	43.7	PHAGE_Salmon_RE_2010_NC_019488
18_GN_21_2229_korea	Global IIa	4	9.9	PHAGE_Phage_Gifsy_2_NC_010393
19_JB_21_3443_korea	Global IIa	1	43.7	PHAGE_Salmon_RE_2010_NC_019488
19_JB_21_3443_korea	Global IIa	2	25.7	PHAGE_Shigel_POCJ13_NC_025434
19_JB_21_3443_korea	Global IIa	3	9.8	PHAGE_Phage_Gifsy_2_NC_010393
19_JB_21_3443_korea	Global IIa	4	31.1	PHAGE_Phage_Gifsy_2_NC_010393
20_JB_21_3450_korea	Global IIa	1	43.7	PHAGE_Salmon_RE_2010_NC_019488
20_JB_21_3450_korea	Global IIa	2	25.7	PHAGE_Shigel_POCJ13_NC_025434
20_JB_21_3450_korea	Global IIa	3	31.1	PHAGE_Phage_Gifsy_2_NC_010393
20_JB_21_3450_korea	Global IIa	4	9.9	PHAGE_Phage_Gifsy_2_NC_010393
21_GJ_S21_korea	Global IIa	1	25.7	PHAGE_Shigel_Stx_NC_029120
21_GJ_S21_korea	Global IIa	2	31.1	PHAGE_Phage_Gifsy_2_NC_010393
21_GJ_S21_korea	Global IIa	3	9.9	PHAGE_Phage_Gifsy_2_NC_010393
21_GJ_S21_korea	Global IIa	4	43.7	PHAGE_Salmon_RE_2010_NC_019488
22_GJ_S22_korea	Global IIa	1	25.7	PHAGE_Shigel_POCJ13_NC_025434
22_GJ_S22_korea	Global IIa	2	31.1	PHAGE_Phage_Gifsy_2_NC_010393
22_GJ_S22_korea	Global IIa	3	9.9	PHAGE_Phage_Gifsy_2_NC_010393
22_GJ_S22_korea	Global IIa	4	43.7	PHAGE_Salmon_RE_2010_NC_019488
23PH-SE10_korea	Global IIa	1	25.7	PHAGE_Shigel_Stx_NC_029120
23PH-SE10_korea	Global IIa	2	31.1	PHAGE_Phage_Gifsy_2_NC_010393
23PH-SE10_korea	Global IIa	3	9.9	PHAGE_Phage_Gifsy_2_NC_010393
23PH-SE10_korea	Global IIa	4	43.7	PHAGE_Salmon_RE_2010_NC_019488
23PH-SE11_korea	Global IIa	1	25.7	PHAGE_Shigel_Stx_NC_029120
23PH-SE11_korea	Global IIa	2	31.1	PHAGE_Phage_Gifsy_2_NC_010393
23PH-SE11_korea	Global IIa	3	9.9	PHAGE_Phage_Gifsy_2_NC_010393
23PH-SE11_korea	Global IIa	4	43.7	PHAGE_Salmon_RE_2010_NC_019488
23PH-SE12_korea	Global IIa	1	43.7	PHAGE_Salmon_RE_2010_NC_019488
23PH-SE12_korea	Global IIa	2	25.7	PHAGE_Shigel_POCJ13_NC_025434
23PH-SE12_korea	Global IIa	3	31.1	PHAGE_Phage_Gifsy_2_NC_010393
23PH-SE12_korea	Global IIa	4	9.9	PHAGE_Phage_Gifsy_2_NC_010393
23PH-SE13_korea	Global IIa	1	9.8	PHAGE_Phage_Gifsy_2_NC_010393
23PH-SE13_korea	Global IIa	2	31.1	PHAGE_Phage_Gifsy_2_NC_010393
23PH-SE13_korea	Global IIa	3	25.7	PHAGE_Shigel_Stx_NC_029120
23PH-SE13_korea	Global IIa	4	43.7	PHAGE_Salmon_RE_2010_NC_019488
23PH-SE14F_korea	Global IIa	1	31.1	PHAGE_Phage_Gifsy_2_NC_010393
23PH-SE14F_korea	Global IIa	2	25.7	PHAGE_Shigel_Stx_NC_029120
23PH-SE14F_korea	Global IIa	3	43.7	PHAGE_Salmon_RE_2010_NC_019488
23PH-SE14F_korea	Global IIa	4	9.9	PHAGE_Phage_Gifsy_2_NC_010393
23PH-SE1_korea	Global IIa	1	9.8	PHAGE_Phage_Gifsy_2_NC_010393

Strain	Global clade	Region	Region size, kb	most_common_phage_name
23PH-SE1_korea	Global IIa	2	31.1	PHAGE_Phage_Gifsy_2_NC_010393
23PH-SE1_korea	Global IIa	3	25.7	PHAGE_Shigel_Stx_NC_029120
23PH-SE1_korea	Global IIa	4	43.7	PHAGE_Salmon_RE_2010_NC_019488
23PH-SE2_korea	Global IIa	1	25.7	PHAGE_Shigel_POCJ13_NC_025434
23PH-SE2_korea	Global IIa	2	31.1	PHAGE_Phage_Gifsy_2_NC_010393
23PH-SE2_korea	Global IIa	3	9.9	PHAGE_Phage_Gifsy_2_NC_010393
23PH-SE2_korea	Global IIa	4	24.2	PHAGE_Salmon_RE_2010_NC_019488
23PH-SE2_korea	Global IIa	5	18.9	PHAGE_Salmon_RE_2010_NC_019488
23PH-SE3_korea	Global IIa	1	9.8	PHAGE_Phage_Gifsy_2_NC_010393
23PH-SE3_korea	Global IIa	2	31.1	PHAGE_Phage_Gifsy_2_NC_010393
23PH-SE3_korea	Global IIa	3	25.7	PHAGE_Shigel_POCJ13_NC_025434
23PH-SE3_korea	Global IIa	4	43.7	PHAGE_Salmon_RE_2010_NC_019488
23PH-SE4_korea	Global IIa	1	9.8	PHAGE_Phage_Gifsy_2_NC_010393
23PH-SE4_korea	Global IIa	2	31.1	PHAGE_Phage_Gifsy_2_NC_010393
23PH-SE4_korea	Global IIa	3	25.7	PHAGE_Shigel_Stx_NC_029120
23PH-SE4_korea	Global IIa	4	43.7	PHAGE_Salmon_RE_2010_NC_019488
23PH-SE5_korea	Global IIa	1	9.8	PHAGE_Phage_Gifsy_2_NC_010393
23PH-SE5_korea	Global IIa	2	31.1	PHAGE_Phage_Gifsy_2_NC_010393
23PH-SE5_korea	Global IIa	3	25.7	PHAGE_Shigel_POCJ13_NC_025434
23PH-SE5_korea	Global IIa	4	25.2	PHAGE_Salmon_RE_2010_NC_019488
23PH-SE5_korea	Global IIa	5	17	PHAGE_Salmon_RE_2010_NC_019488
23PH-SE6_korea	Global IIa	1	9.8	PHAGE_Phage_Gifsy_2_NC_010393
23PH-SE6_korea	Global IIa	2	31.1	PHAGE_Phage_Gifsy_2_NC_010393
23PH-SE6_korea	Global IIa	3	25.7	PHAGE_Shigel_POCJ13_NC_025434
23PH-SE6_korea	Global IIa	4	43	PHAGE_Salmon_RE_2010_NC_019488
23PH-SE7_korea	Global IIa	1	43.7	PHAGE_Salmon_RE_2010_NC_019488
23PH-SE7_korea	Global IIa	2	25.7	PHAGE_Shigel_POCJ13_NC_025434
23PH-SE7_korea	Global IIa	3	31.1	PHAGE_Phage_Gifsy_2_NC_010393
23PH-SE7_korea	Global IIa	4	9.9	PHAGE_Phage_Gifsy_2_NC_010393
23PH-SE8_korea	Global IIa	1	25.7	PHAGE_Shigel_Stx_NC_029120
23PH-SE8_korea	Global IIa	2	31.1	PHAGE_Phage_Gifsy_2_NC_010393
23PH-SE8_korea	Global IIa	3	9.9	PHAGE_Phage_Gifsy_2_NC_010393
23PH-SE8_korea	Global IIa	4	43.7	PHAGE_Salmon_RE_2010_NC_019488
23PH-SE9_korea	Global IIa	1	25.7	PHAGE_Shigel_POCJ13_NC_025434
23PH-SE9_korea	Global IIa	2	31.1	PHAGE_Phage_Gifsy_2_NC_010393
23PH-SE9_korea	Global IIa	3	9.9	PHAGE_Phage_Gifsy_2_NC_010393
23PH-SE9_korea	Global IIa	4	43.7	PHAGE_Salmon_RE_2010_NC_019488
26_CN_BR_korea	Global IIa	1	43.7	PHAGE_Salmon_RE_2010_NC_019488
26_CN_BR_korea	Global IIa	2	25.7	PHAGE_Shigel_POCJ13_NC_025434
26_CN_BR_korea	Global IIa	3	31.1	PHAGE_Phage_Gifsy_2_NC_010393
26_CN_BR_korea	Global IIa	4	9.9	PHAGE_Phage_Gifsy_2_NC_010393
27_CN_GJ_korea	Global IIa	1	25.7	PHAGE_Shigel_Stx_NC_029120
27_CN_GJ_korea	Global IIa	2	31.1	PHAGE_Phage_Gifsy_2_NC_010393
27_CN_GJ_korea	Global IIa	3	9.9	PHAGE_Phage_Gifsy_2_NC_010393
27_CN_GJ_korea	Global IIa	4	35.9	PHAGE_Salmon_RE_2010_NC_019488
SAL_AB8575AA_AS	Global IIa	2	16.5	PHAGE_Shigel_POCJ13_NC_025434(4),PHAGE_Shigel_Stx_NC_029120(4)
SAL_AB8575AA_AS	Global IIa	3	31.1	PHAGE_Phage_Gifsy_2_NC_010393(17),PHAGE_Phage_Gifsy_1_NC_010392(11)
SAL_AB8575AA_AS	Global IIa	4	43.7	PHAGE_Salmon_RE_2010_NC_019488(39),PHAGE_Salmon_Fels_2_NC_010463(34)
SAL_EC2476AA_AS	Global IIa	1	43.7	PHAGE_Salmon_RE_2010_NC_019488(39),PHAGE_Salmon_Fels_2_NC_010463(34)

Strain	Global clade	Region	Region size, kb	most_common_phage_name
SAL_EC2476AA_AS	Global IIa	2	16.5	PHAGE_Shigel_Stx_NC_029120(4),PHAGE_Shigel_POCJ13_NC_025434(4)
SAL_EC2476AA_AS	Global IIa	3	31.1	PHAGE_Phage_Gifsy_2_NC_010393(17),PHAGE_Phage_Gifsy_1_NC_010392(11)
SAL_JC5401AA_AS	Global IIa	1	43.7	PHAGE_Salmon_RE_2010_NC_019488(39),PHAGE_Salmon_Fels_2_NC_010463(34)
SAL_JC5401AA_AS	Global IIa	2	31.1	PHAGE_Phage_Gifsy_2_NC_010393(17),PHAGE_Phage_Gifsy_1_NC_010392(11)
SAL_KC0777AA_AS	Global IIa	1	31.1	PHAGE_Phage_Gifsy_2_NC_010393(17),PHAGE_Phage_Gifsy_1_NC_010392(11)
SAL_KC0777AA_AS	Global IIa	2	9.8	PHAGE_Phage_Gifsy_2_NC_010393(5),PHAGE_Entero_HK225_NC_019717(2)
SAL_KC0777AA_AS	Global IIa	3	43.7	PHAGE_Salmon_RE_2010_NC_019488(39),PHAGE_Salmon_Fels_2_NC_010463(34)
SAL_KC0826AA_AS	Global IIa	1	9.8	PHAGE_Phage_Gifsy_2_NC_010393(5),PHAGE_Entero_HK225_NC_019717(2)
SAL_KC0826AA_AS	Global IIa	2	31.1	PHAGE_Phage_Gifsy_2_NC_010393(17),PHAGE_Phage_Gifsy_1_NC_010392(11)
SAL_KC0826AA_AS	Global IIa	3	15.4	PHAGE_Shigel_POCJ13_NC_025434(4),PHAGE_Shigel_Stx_NC_029120(4)
SAL_KC0826AA_AS	Global IIa	4	43.7	PHAGE_Salmon_RE_2010_NC_019488(39),PHAGE_Salmon_Fels_2_NC_010463(34)
SAL_NC6353AA_AS	Global IIa	1	43.7	PHAGE_Salmon_RE_2010_NC_019488(39),PHAGE_Salmon_Fels_2_NC_010463(34)
SAL_NC6353AA_AS	Global IIa	2	16.5	PHAGE_Shigel_Stx_NC_029120(4),PHAGE_Shigel_POCJ13_NC_025434(4)
SAL_NC6353AA_AS	Global IIa	3	31.1	PHAGE_Phage_Gifsy_2_NC_010393(17),PHAGE_Phage_Gifsy_1_NC_010392(11)
SAL_PB8568AA_AS	Global IIa	1	16.5	PHAGE_Shigel_POCJ13_NC_025434(4),PHAGE_Shigel_Stx_NC_029120(4)
SAL_PB8568AA_AS	Global IIa	2	31.1	PHAGE_Phage_Gifsy_2_NC_010393(17),PHAGE_Phage_Gifsy_1_NC_010392(11)
SAL_PB8568AA_AS	Global IIa	3	9.8	PHAGE_Phage_Gifsy_2_NC_010393(5),PHAGE_Entero_HK225_NC_019717(2)
SAL_PB8568AA_AS	Global IIa	4	43.7	PHAGE_Salmon_RE_2010_NC_019488(39),PHAGE_Salmon_Fels_2_NC_010463(34)
SAL_SC1704AA_AS	Global IIa	1	43.7	PHAGE_Salmon_RE_2010_NC_019488(39),PHAGE_Salmon_Fels_2_NC_010463(34)
SAL_SC1704AA_AS	Global IIa	2	16.7	PHAGE_Shigel_Stx_NC_029120(4),PHAGE_Shigel_POCJ13_NC_025434(4)
SAL_SC1704AA_AS	Global IIa	3	31.1	PHAGE_Phage_Gifsy_2_NC_010393(17),PHAGE_Phage_Gifsy_1_NC_010392(11)
SAL_SC8599AA_AS	Global IIa	1	16.5	PHAGE_Shigel_Stx_NC_029120(4),PHAGE_Shigel_POCJ13_NC_025434(4)
SAL_SC8599AA_AS	Global IIa	2	31.1	PHAGE_Phage_Gifsy_2_NC_010393(17),PHAGE_Phage_Gifsy_1_NC_010392(11)
SAL_SC8599AA_AS	Global IIa	3	43.7	PHAGE_Salmon_RE_2010_NC_019488(39),PHAGE_Salmon_Fels_2_NC_010463(34)
SAL_UC5098AA_AS	Global IIa	1	31.1	PHAGE_Phage_Gifsy_2_NC_010393(17),PHAGE_Phage_Gifsy_1_NC_010392(11)
SAL_UC5098AA_AS	Global IIa	2	15.4	PHAGE_Shigel_POCJ13_NC_025434(4),PHAGE_Shigel_Stx_NC_029120(4)
SAL_UC5098AA_AS	Global IIa	3	43.7	PHAGE_Salmon_RE_2010_NC_019488(39),PHAGE_Salmon_Fels_2_NC_010463(34)
SAL_VB8975AA_AS	Global IIa	1	31.1	PHAGE_Phage_Gifsy_2_NC_010393(17),PHAGE_Phage_Gifsy_1_NC_010392(11)
SAL_VB8975AA_AS	Global IIa	2	15.4	PHAGE_Shigel_Stx_NC_029120(4),PHAGE_Shigel_POCJ13_NC_025434(4)
SAL_VB8975AA_AS	Global IIa	3	43.7	PHAGE_Salmon_RE_2010_NC_019488(39),PHAGE_Salmon_Fels_2_NC_010463(34)
SAL_VC5187AA_AS	Global IIa	1	31.1	PHAGE_Phage_Gifsy_2_NC_010393(17),PHAGE_Phage_Gifsy_1_NC_010392(11)
SAL_VC5187AA_AS	Global IIa	2	43.7	PHAGE_Salmon_RE_2010_NC_019488(39),PHAGE_Salmon_Fels_2_NC_010463(34)
SAL_VC6111AA_AS	Global IIa	1	14.2	PHAGE_Phage_Gifsy_1_NC_010392(2),PHAGE_Pseudo_F116_NC_006552(2)
SAL_VC6111AA_AS	Global IIa	2	31.1	PHAGE_Phage_Gifsy_2_NC_010393(17),PHAGE_Phage_Gifsy_1_NC_010392(11)
SAL_VC6111AA_AS	Global IIa	3	15.4	PHAGE_Shigel_Stx_NC_029120(4),PHAGE_Shigel_POCJ13_NC_025434(4)
SAL_VC6111AA_AS	Global IIa	4	43.7	PHAGE_Salmon_RE_2010_NC_019488(39),PHAGE_Salmon_Fels_2_NC_010463(34)
SAL_WB3333AA_AS	Global IIa	1	31.1	PHAGE_Phage_Gifsy_2_NC_010393(17),PHAGE_Phage_Gifsy_1_NC_010392(11)
SAL_WB3333AA_AS	Global IIa	2	15.4	PHAGE_Shigel_Stx_NC_029120(4),PHAGE_Shigel_POCJ13_NC_025434(4)
SAL_WB3333AA_AS	Global IIa	3	43.7	PHAGE_Salmon_RE_2010_NC_019488(39),PHAGE_Salmon_Fels_2_NC_010463(34)
SAL_XC6397AA_AS	Global IIa	1	43.7	PHAGE_Salmon_RE_2010_NC_019488(39),PHAGE_Salmon_Fels_2_NC_010463(34)
SAL_XC6397AA_AS	Global IIa	2	31.1	PHAGE_Phage_Gifsy_2_NC_010393(17),PHAGE_Phage_Gifsy_1_NC_010392(11)
SAL_XC6397AA_AS	Global IIa	3	15.4	PHAGE_Shigel_POCJ13_NC_025434(4),PHAGE_Shigel_Stx_NC_029120(4)
SAL_YC1440AA_AS	Global IIa	1	15.4	PHAGE_Shigel_POCJ13_NC_025434(4),PHAGE_Shigel_Stx_NC_029120(4)
SAL_YC1440AA_AS	Global IIa	2	43.7	PHAGE_Salmon_RE_2010_NC_019488(39),PHAGE_Salmon_Fels_2_NC_010463(34)
SAL_YC1440AA_AS	Global IIa	3	31.1	PHAGE_Phage_Gifsy_2_NC_010393(17),PHAGE_Phage_Gifsy_1_NC_010392(11)
4_ChickenFarm_korea	Global IIa	1	24.2	PHAGE_Phage_Gifsy_2_NC_010393
4_ChickenFarm_korea	Global IIa	2	43.7	PHAGE_Salmon_RE_2010_NC_019488
4_ChickenFarm_korea	Global IIa	3	25.7	PHAGE_Shigel_Stx_NC_029120
5_ChickenFarm_korea	Global IIa	1	25.7	PHAGE_Shigel_POCJ13_NC_025434

Strain	Global clade	Region	Region size, kb	most_common_phage_name
5_ChickenFarm_korea	Global IIa	2	9.9	PHAGE_Phage_Gifsy_2_NC_010393
5_ChickenFarm_korea	Global IIa	3	31.1	PHAGE_Phage_Gifsy_2_NC_010393
5_ChickenFarm_korea	Global IIa	4	43.7	PHAGE_Salmon_RE_2010_NC_019488
CP110220_korea	Global IIa	1	43.7	PHAGE_Salmon_RE_2010_NC_019488
CP110220_korea	Global IIa	2	25.7	PHAGE_Shigel_POCJ13_NC_025434
CP110220_korea	Global IIa	3	31.1	PHAGE_Phage_Gifsy_2_NC_010393
CP110220_korea	Global IIa	4	9.9	PHAGE_Phage_Gifsy_2_NC_010393
SAL_AB8575AA_AS	Global IIa	1	17.2	PHAGE_Phage_Gifsy_2_NC_010393(5),PHAGE_Salmon_Fels_1_NC_010391(2)
23_GG_13_4945_korea	Global III	1	25.7	PHAGE_Shigel_POCJ13_NC_025434
23_GG_13_4945_korea	Global III	2	31.1	PHAGE_Phage_Gifsy_2_NC_010393
23_GG_13_4945_korea	Global III	3	9.8	PHAGE_Phage_Gifsy_2_NC_010393
23_GG_13_4945_korea	Global III	4	43.7	PHAGE_Salmon_RE_2010_NC_019488
24_BS_14_3792_korea	Global III	1	43.7	PHAGE_Salmon_RE_2010_NC_019488
24_BS_14_3792_korea	Global III	2	9.8	PHAGE_Phage_Gifsy_2_NC_010393
24_BS_14_3792_korea	Global III	3	31.1	PHAGE_Phage_Gifsy_2_NC_010393
24_BS_14_3792_korea	Global III	4	25.7	PHAGE_Shigel_Stx_NC_029120
25_DJ_14_5950_korea	Global I	1	54	PHAGE_Salmon_118970_sal3_NC_031940
25_DJ_14_5950_korea	Global I	2	25.7	PHAGE_Shigel_POCJ13_NC_025434
25_DJ_14_5950_korea	Global I	3	31.1	PHAGE_Phage_Gifsy_2_NC_010393
25_DJ_14_5950_korea	Global I	4	9.9	PHAGE_Phage_Gifsy_2_NC_010393

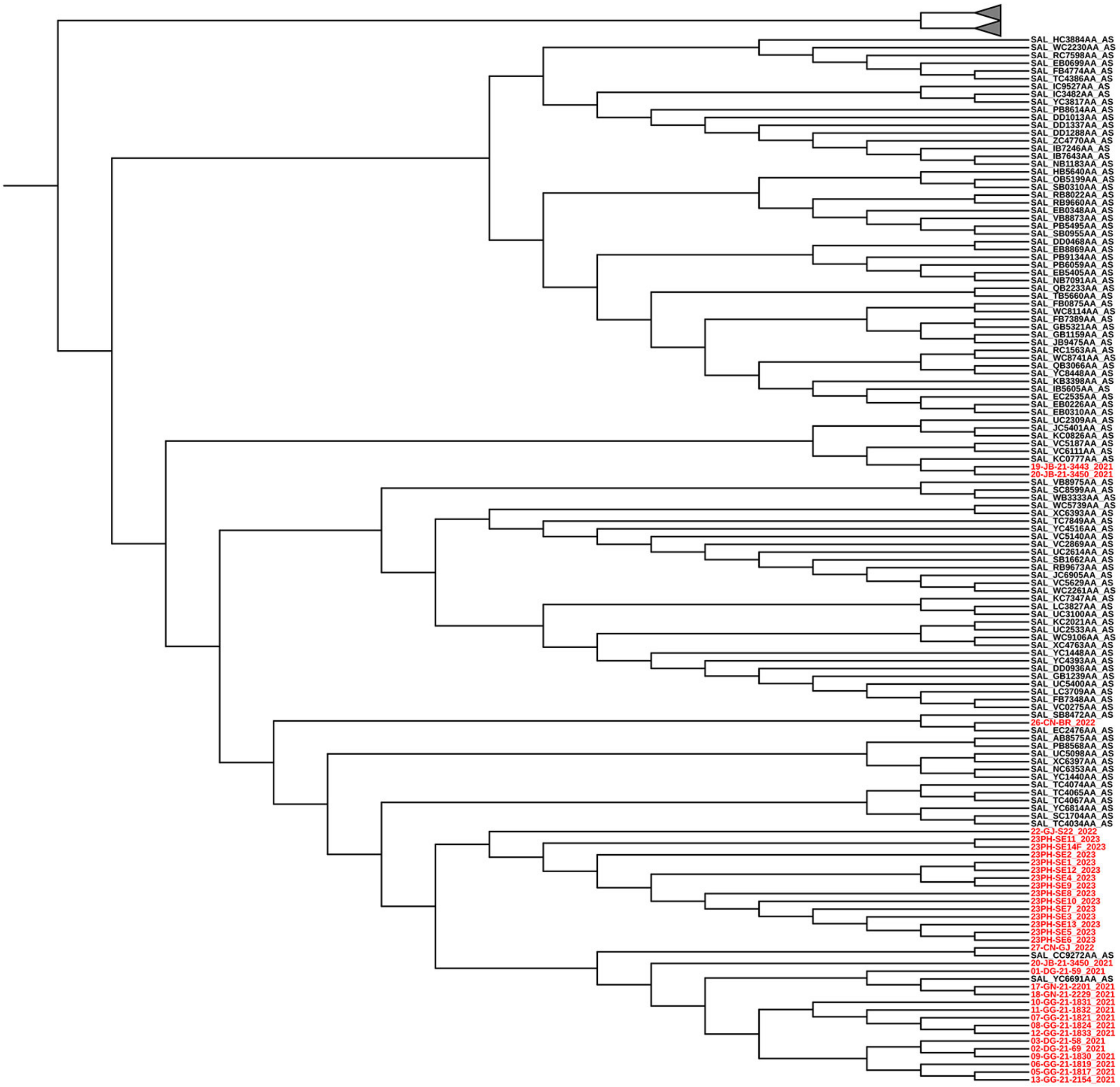

Appendix 1 Figure. Phylogenetic analysis of the core-genome multilocus sequence typing HC5_2301 cluster (n = 3,609) and *Salmonella* Enteritidis isolates from this study (n = 38). Neighbor-joining phylogenetic tree was constructed by using Mashtree (https://github.com/lskatz/mashtree). Isolates from this study are highlighted in red. Tree not drawn to scale.
